# Prevalence of prediabetes, and diabetes in Chandigarh and Panchkula region based on glycated haemoglobin and Indian diabetes risk score

**DOI:** 10.1002/edm2.162

**Published:** 2020-11-11

**Authors:** Saurabh Kumar, Akshay Anand, Raghuram Nagarathna, Navneet Kaur, Madhava Sai Sivapuram, Viraaj Pannu, Deepak Kumar Pal, Neeru Malik, Amit Kumar Singh, Hongasandra Ramarao Nagendra

**Affiliations:** ^1^ Neuroscience Research Lab Department of Neurology Postgraduate Institute of Medical Education and Research (PGIMER) Chandigarh India; ^2^ Swami Vivekananda Yoga Anusandhana Samsthana (S‐VYASA) Bengaluru India; ^3^ Department of Physical Education Panjab University Chandigarh India; ^4^ Department of General Medicine Dr. Pinnamaneni Siddhartha Institute of Medical Sciences and Research Foundation Chinna‐Avutapalli India; ^5^ Government Medical College and Hospital Sector‐32 Chandigarh India; ^6^ Dev Samaj College of Education Chandigarh India

**Keywords:** blood glucose, diabetes, glycated haemoglobin, Indian diabetes risk score

## Abstract

There is a rapid increase in the prevalence of diabetes in India. We wanted to review the status of prediabetes and diabetes in the combined population of Chandigarh and Panchkula region based on both Indian Diabetes Risk Score (IDRS) and Glycated Haemoglobin (HbA1c). A total of 1215 subjects were recruited during the screening process, out of which 444 i subjects have been analysed for the current study on the basis of high risk for IDRS (≥60) and their known diabetes status. This study included 431 subjects having high risk for IDRS (≥60) and 13 known subjects with diabetes (IDRS < 60) which were further analysed for biochemical and anthropometric parameters. The prevalence of diabetes was found to be 12.67% and prediabetes 11.69% in the combined population of Chandigarh and Panchkula. There was an increased level of fasting blood glucose (183.12 ± 68.61), postprandial blood glucose (262.57 ± 96.92), triglyceride (193.84 ± 119.88), very low‐density lipoprotein (VLDL) (34.87 ± 15.42) and High Density Lipoprotein(HDL) (4.61 ± 1.39) in the said diabetes population. Mean HDL was found to be decreased in subjects having diabetes. Glucose‐induced lipid intolerance study revealed significant alteration in triglyceride, HDL and VLDL. The study has revealed that high prevalence of diabetes in the sampled population when compared with the national average of 8.8%.

## Graphical abstract

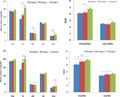

## 1 INTRODUCTION

Percentage of glycated haemoglobin (HbA1c) is used as an important biochemical parameter to assess past three month's blood glucose status.^1^ Factors affecting HbA1c level include blood sugar concentration, Red Blood Cell (RBC) duration to varying concentrations and quantity of RBC. In 2009, American Diabetes Association (ADA) and, in 2011 World Health Organization (WHO) recommended that HbA1c as a tool for the screening, diagnosis and monitoring of diabetes.^2^ According to ADA guidelines, the recommended values for the diagnosis of diabetes includes HbA1c ≥ 6.5% (48 mmol/mol), fasting sugar ≥ 126 mg/dL (7.0 mmol/L) and 2‐hr postprandial level ≥ 200 mg/dL (11.1 mmol/L).^2^ At cut‐off value of 6.5% HbA1c demonstrated the sensitivity of 44% and specificity of 99% in adult population (69.4 ± 11.1 years).^3^ For prediabetes and healthy individuals, the recommended HbA1c value is 5.7%‐6.4% and <5.7%, respectively.^4^ A 6‐year diabetes prediction study showed that at 5.7% HbA1c cut‐point, the sensitivity was 66% whilst the specificity was 88%.^5^ Similarly, a study from National Health and Nutrition Examination Survey (NHANES) demonstrated the sensitivity of HbA1c to be 39%‐45% and specificity to be 81%‐91% for 5.7% HbA1c. Another study predicted the association of 5.7% HbA1c with high risk for diabetes. Therefore, a value of 5.7%‐6.4% is considered reasonable for identification of individuals within prediabetes.^6^ HbA1c acts as a dependable method to detect chronic hyperglycaemia and the risk of developing other diabetes complications in long term. Studies suggest that the increased level of HbA1c acts as an independent risk factor for complications like stroke and coronary heart disease in individuals with or without diabetes.^7^ In India, diabetes mellitus (DM) is amongst the leading health problem which profoundly affects the health budget due to the comorbidities associated with it.^8^ A total of 415 million individuals have been reported to be affected with diabetes which is further expected to rise up to 642 million by the end of 2040.^9^ About 75% of the low/middle income countries will be affected. This drastic increase in the number of affected individuals with diabetes poses a threat to the productivity and economy of a developing country. It was estimated that the annual expenditure on diabetes costs about USD 760 billion which is 10% of the total annual global health expenditure. India has estimated 72, 946, 000 diabetes cases (with 8.8% prevalence in adults) is the second largest after China.^10^ In India, the average cost per person on diabetes was USD 67.98 in 2012.^11^ According to a study published in Lancet, the number of individuals with diabetes was drastically increased in India from 1980 to 2016. In 1990, there were 26.0 million people suffering with diabetes in India, which increased to 65.0 million in 2016.^12^ The prevalence of diabetes in India varies across various states, ranging between 5% to 17% with highest prevalence in southern states and in urban areas.^9^ A survey report (conducted between 2015‐19) published by Ministry of Health and Family Welfare reports the prevalence of diabetes to be 11.8% for these 4 years. This report has revealed the prevalence of known diabetes cases to be 8%, whereas the prevalence of new cases was 3.8%.^13^ The estimates of prevalence and identification of the individuals with high risk for diabetes are important for the planning. The identification of these high‐risk individuals are equally important, and this can only be achieved if they are identified at transition state or before that. Prediabetes can be considered as the transition state between a healthy and a diabetes individual. Prediabetes, also called intermediate hyperglycaemia, is a condition in which the serum blood glucose levels are higher than the normal levels, but not enough to cause diabetes. According to ADA, the cut‐off level for prediabetes is 5.7%‐6.4%. Prediabetes is linked with the abnormalities in the form of insulin resistance and β‐cell dysfunction which starts before glucose changes are measurable. It is estimated that there will be >470 million people with prediabetes in 2030. The conversion rate of prediabetes to diabetes is around 5%‐10%.^14^

Furthermore, by identification of prediabetes in the population, the threat of conversion from prediabetes into diabetes can be reduced.^15^ The Indian Diabetes Risk Score (IDRS) is a method developed by Mohan et al in 2005 to analyse the risk of prediabetes/diabetes at mass level.^16^ IDRS considers four parameters: age, family history (father or mother), physical activity and abdominal obesity (waist circumference). Risk assessment by IDRS involves 3 categories: score < 30 (low risk), 30‐50 (moderate risk) and ≥60 (high risk). We performed a cross‐sectional study in 2 regions of North West India population (Chandigarh and Panchkula) based on IDRS in order to explore the prediabetes and diabetes individuals in the community further validated on the basis of HbA1c levels. Individuals with high risk (IDRS score ≥ 60) were selected for the analysis. The specificity of IDRS score ≥ 60 was 60.1%, whereas the sensitivity was 72.5%.^17^

## 2 METHODOLOGY

### 2.1 Study design

**Figure 1:**
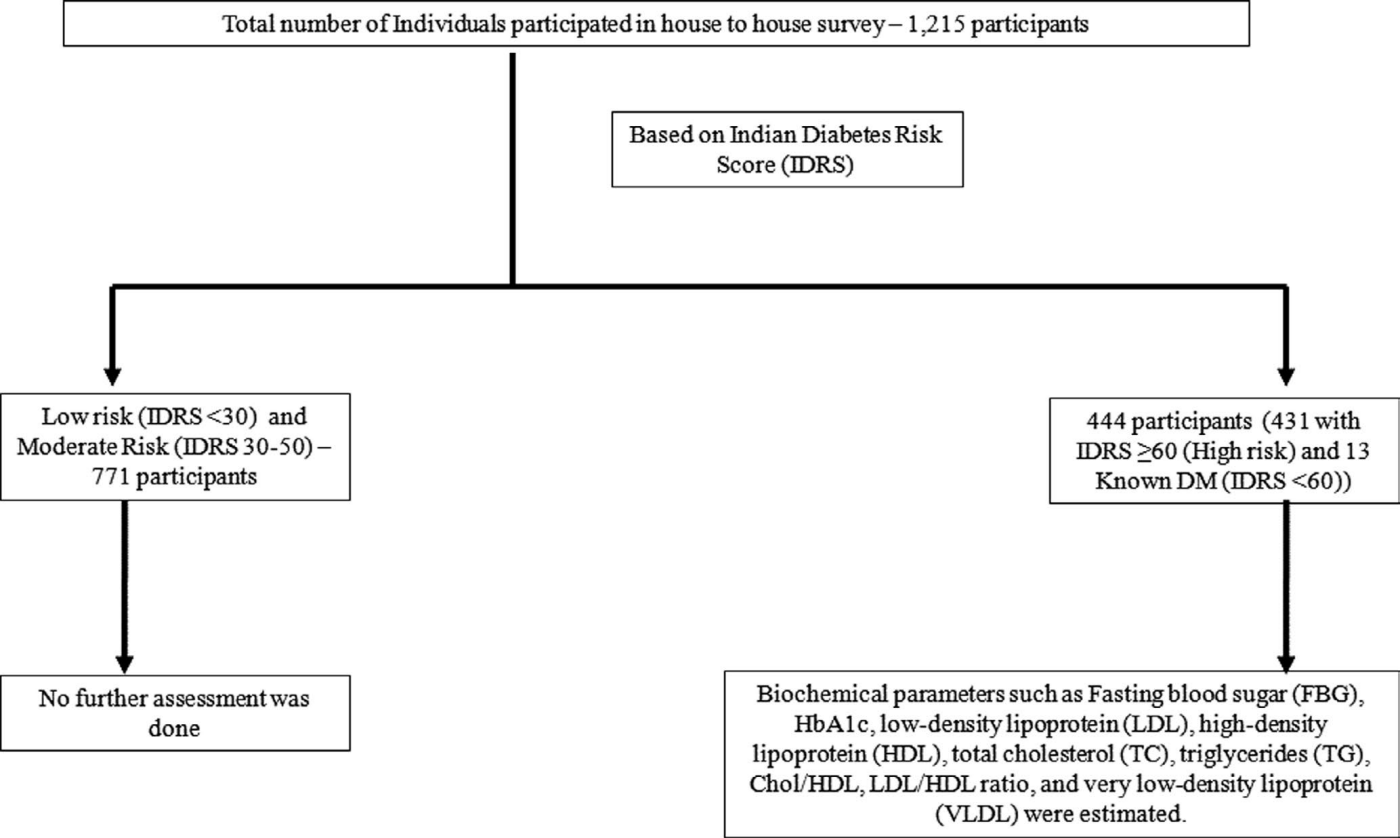
Schematic representation of recruitment of individuals

This study was a part of *Niyantrita Madhumeha Bharata* (NMB) programme, in which 29 Indian States and 7 Union Territories (UTs) were screened. It was a multi‐level cluster randomized controlled trial. However, the data presented in this study are of two regions of North West India i.e. Chandigarh (Union Territory), and Panchkula (District). For sample size calculation, we referred to Diabetes Community Lifestyle Improvement Program (D‐CLIP) study published in diabetes care.^18^ The details are published in our recent publications.^19^, ^20^ As a part of this national programme, house‐to‐house screening was carried out by trained volunteers of Indian Yoga Association (IYA). A two‐page questionnaire was used which comprises of the personal information about name, age, family history of diabetes, waist circumference, height and weight, besides collecting the workout information. Based on this, IDRS score was calculated.^17^ Initially, a total of 1215 subjects were recruited for the study, out of which 444 subjects were assessed for the biochemical parameters based on high risk for IDRS (IDRS ≥ 60) and their known diabetes status. The Figure No ‐ 1 shows the schematic of recruitment of an individuals for the study. Kish formula for house‐to‐house screening was not used (Figure 1).

#### 2.1.1 Inclusion and exclusion criteria used for screening was as following

##### Inclusion criteria

- Both male and female participants with diabetes (self‐reported, which was cross verified)
- Subjects were from within the periphery of 10km distance from rural and urban regions.
- Those individuals who showed an IDRS score ≥ 60 (for further recruitment)
- Patients/Subjects who gave their consents for the study.

##### Exclusion criteria

- Age below 18 years
- Patients with cardiac disease and tuberculosis.
- Those who had complex surgeries in past.
- Those who had major illness which may disable an individual having diabetes.
- Individuals with neurological disorders.

### 2.2 Assessments

#### 2.2.1 Biochemical

Participants were called for the special blood collection camp on empty stomach with minimum eight hours of fasting. The blood sampling and subsequent biochemical analysis was carried out by an National Accreditation Board for Testing and Calibration Laboratories (NABL) accredited laboratory. Fasting blood glucose (FBG), HbA1c, low‐density lipoprotein (LDL), high‐density lipoprotein (HDL), total cholesterol (TC), triglycerides (TG), cholesterol/HDL, LDL/HDL ratio and very low‐density lipoprotein (VLDL) were analysed for such fasting samples. An autoanalyser (model 2700/480) (Validation: Beckman Coulter) for the estimation of TC, TG, LDL, HDL and VLDL was used. For HbA1c, we used HPLC‐based technique using the Variant II Turbo machine (Bio‐Rad, Hercules). Postprandial blood glucose (PPG) sample was taken after 2 hours of breakfast and estimated using Mindray Autoanalyzer (BS‐390 model). The methods used for the assessment were certified by the National Glycol Haemoglobin Standardization Program (NGHSP) as detailed in our recent publication.^19^

#### 2.2.2 Anthropometry

.

Height ( Stadiometer) and waist Circimference ( Standard measuring tape) was measured in centimeters. weight. Weights ( digital weighing machine) was measured in kgs.

### 2.3 Ethical statement

Ethical clearance for the study was obtained from the Institutional Ethical Committee of Indian Yoga Association, via letter number: RES/IEC‐IYA/001 dated 16th December 2016. Blood samples of the participants were taken after the written informed consent was signed by them. This study was primarily done by Swami Vivekananda Yoga Anusandhana Samsthana (S‐VYASA) under IYA.

### 2.4 Statistical analysis

The statistical analysis was done by using the IBM SPSS Statistics Version 21. The data are presented as mean, standard deviation (SD) and standard error (SE). Descriptive statistics was performed by one‐way ANOVA. The data were found to be statistically significant at *P* < .05.

## 3 RESULTS

Based on the house‐to‐house screening, 1215 individuals were recruited and screened as per IDRS score as low‐risk, moderate‐risk and high‐risk individuals. Out of 1215, a total of 444 subjects were found to be at high risk (≥60 or known diabetes status) which were further assessed according to HbA1c levels. The final prevalence of diabetes, prediabetes subjects were found to be 12.67% and 11.69%, respectively, as depicted in the Table 1.

**Table 1:** Prevalence of diabetes, and prediabetes in the combined population of Chandigarh and Panchkula

S No.	Population subsets		Number of participants	Prevalence (%)
1.	*Diabetes Population (High‐risk IDRS/Known DM and HbA1c >6.5)		154	12.67
2.	Prediabetes Population (High‐risk IDRS and HbA1c: 5.7 ‐ 6.4)		142	11.69
3.	Healthy Population	High‐risk IDRS and HbA1c ≤ 5.6 ‐ (n = 148) participants	919	75.64
		Low‐ and moderate‐risk IDRS ‐ (n = 771) participants		
	Total		1215	100

* There were total 13 individuals with IDRS score less than 60, but HbA1c ≥ 6.5.

**Figure 2:**
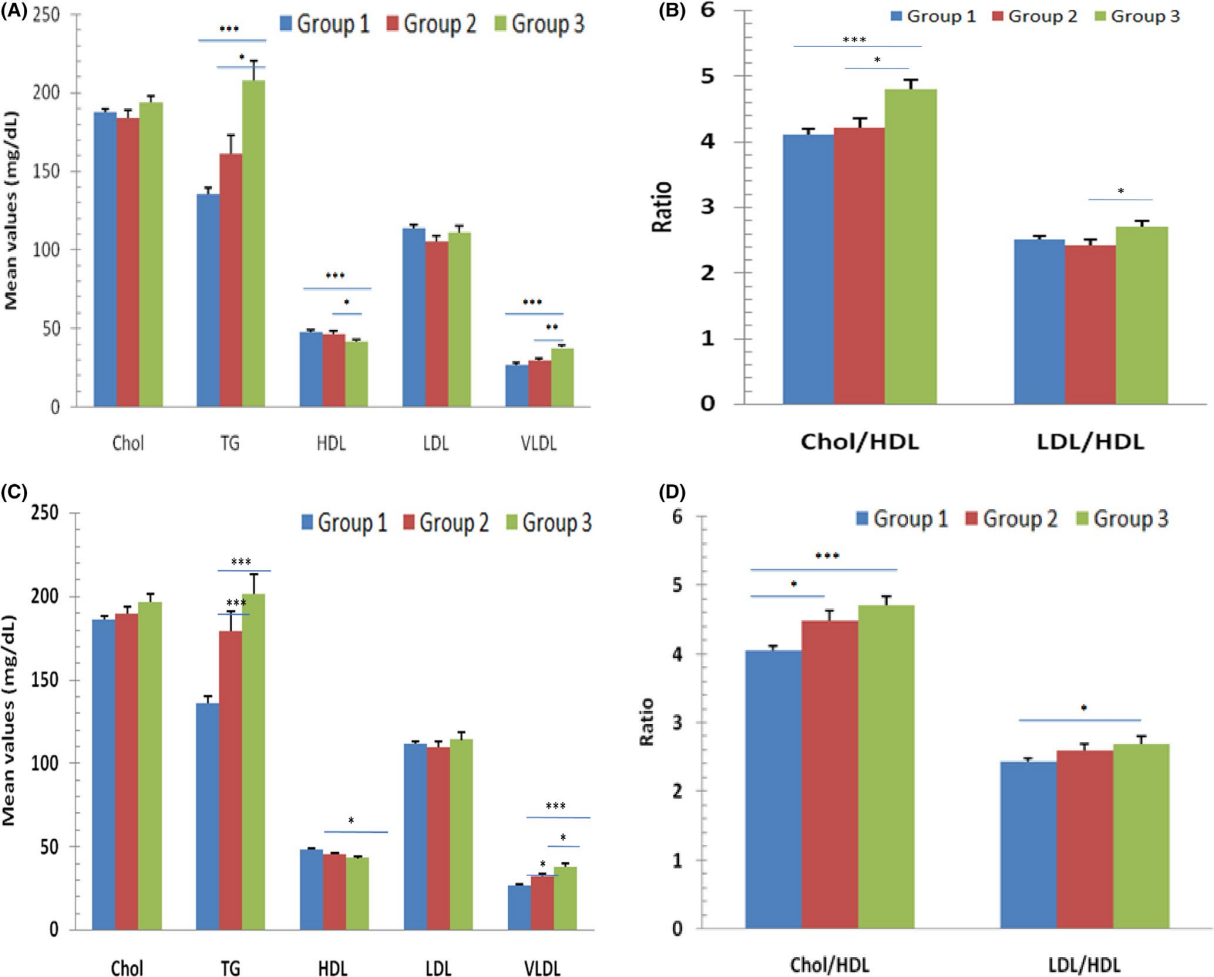
Glucose‐induced lipid profile intolerance: (A & B) determined by postprandial glucose (PPG), considering its specific range (80‐140 mg/dL: Group 1; 141‐220 mg/dL: Group 2; ≥221 mg/dL: Group 3). (C & D) determined by fasting blood glucose (FBG), with specific range (70‐110 mg/dL: Group 1; 111‐160 mg/dL: Group 2; ≥161 mg/dL: Group 3)

Table No 2 summarizes the comparison between the three groups: diabetes, prediabetes and healthy population amongst the high‐risk IDRS individuals (≥60). Out of 444 subject samples analysed, 307 were females and 137 were males. The estimated FBG level was highest in diabetes group (183.12 ± 68.61) than the healthy (93.01 ± 7.80) and prediabetes group (102.27 ± 13.26). Similarly, the mean PPG levels in diabetes group were 262.57 ± 96.92, which was higher than healthy (96.53 ± 17.14) and prediabetes group (121.75 ± 36.32). We found TG levels were more (193.84 ± 119.88) in diabetes group than the other two group used in the present study. VLDL was also found to be increased in individuals with diabetes (34.87 ± 15.42). No much difference in mean TC was observed in three groups. Mean TC for diabetes group was 191.58 ± 43.06, for prediabetes it was 190.99 ± 35.46, and for group with HbA1c ≤ 5.6 it was 182.93 ± 36.88. Amongst the high‐risk IDRS individuals, the mean IDRS was found to be 71.15 ± 10.20 for healthy group, 76.41 ± 9.99 for prediabetes and 75.19 ± 14.69 for diabetes. Good cholesterol (HDL) was found to be reduced in diabetes group (43.52 ± 9.91), in comparison with the prediabetes (47.46 ± 12.78) and healthy group (48.69 ± 13.04). Apparently, the anthropometric parameters, namely BMI (29.30 ± 4.46) and weight (72.58 ± 11.47), were highest amongst the prediabetes subjects. We measured the glucose‐induced lipid intolerance by estimating different lipid parameters with respect to FBG and PPG, considering the reference range of FBG as 70‐110 mg/dL and a range of 80‐140 mg/dL for PPG.^21^ We found that for PPG and FBG, parameters like triglyceride and VLDL showed significant increased (*P* < .05) in lipid intolerance, whereas HDL showed significant decrease (Figure 2) (Table S1).

**Table 2:** Comparative analysis of various physiological and biochemical parameters in Healthy, Prediabetes and Diabetes individuals with respect to HbA1c

Subgroups (HbA1c)	Total number (n)	Healthy (≤ 5.6) (Mean ± SD) (SE)	Prediabetes (5.7‐6.4) (Mean ± SD) (SE)	Diabetes (≥6.5) (Mean ± SD) (SE)	*Significance (*P*‐Value)
(Female/Male)	444 (307/137)	148 (111/37)	142 (106/36)	154 (90/64)	‐
Age (years)		45.77 ± 10.20 (1.07)	51.95 ± 10.78 (1.15)	53.79 ± 9.81 (1.02)	**‐**
Weight (Kg)	444	69.33 ± 14.10 (1.16)	72.58 ± 11.47 (0.96)	70.29 ± 12.76 (1.03)	‐
BMI (Kg/m^2^)	444	27.88 ± 5.94 (0.49)	29.30 ± 4.46 (0.37)	27.58 ± 4.80 (0.39)	.009
IDRS	444	71.15 ± 10.20 (0.84)	76.41 ± 9.99 (0.84)	75.19 ± 14.69 (1.18)	**<.001**
Fasting Blood Glucose (FBG) (mg/dL)	444	93.01 ± 7.80 (0.64)	102.27 ± 13.26 (1.11)	183.12 ± 68.61 (5.53)	**<.001**
Postprandial Glucose (PPG) (mg/dL)	419	96.53 ± 17.14 (1.49)	121.75 ± 36.32 (3.06)	262.57 ± 96.92 (8.05)	**<.001**
Cholesterol (mg/dL)	444	182.93 ± 36.88 (3.03)	190.99 ± 35.46 (2.98)	191.58 ± 43.06 (3.47)	.100
Triglyceride (mg/dL)	444	134.92 ± 71.39 (5.87)	140.13 ± 67.52 (5.67)	193.84 ± 119.88 (9.66)	**<.001**
HDL (mg/dL)	444	48.69 ± 13.04 (1.07)	47.46 ± 12.78 (1.07)	43.52 ± 9.91 (0.79)	.001
LDL (mg/dL)	441	109.01 ± 29.06 (2.40)	115.50 ± 29.78 (2.49)	109.64 ± 34.96 (2.83)	.156
Cholesterol/HDL	444	3.96 ± 1.13 (0.09)	4.22 ± 1.14 (0.09)	4.61 ± 1.39 (0.11)	**<.001**
LDL/HDL	443	2.38 ± 0.73 (0.06)	2.57 ± 0.86 (0.07)	2.60 ± 0.93 (0.07)	.045
VLDL	435	26.79 ± 13.36 (1.10)	27.51 ± 12.07 (1.02)	34.87 ± 15.42 (1.27)	**<.001**

P values which are considered statistically significant and indicated in bold.

* Between group analysis (ANOVA); *P* < .05.

## 4 DISCUSSION

In the present study, we have analysed the prevalence of prediabetes, and diabetes subjects based on IDRS score and HbA1c levels. Our study is based on IDRS assessment to diagnose diabetes and prediabetes in the target population. There are very few IDRS‐based studies in the target population (Chandigarh and Panchkula). In previously published study very low sample size (n = 155) was used.^22^ Therefore, we wanted to study the prevalence of diabetes and prediabetes in larger sample size (n = 444). According to present study, the prevalence of prediabetes and diabetes was 11.69% and 12.67%, respectively. Clinical parameters such as FBG and PPG are considered as standard parameters for glycaemic index.^23^ Therefore, considering these two parameters, we analysed the glucose‐induced lipid intolerance. We found significant alteration in case of triglyceride (*increased*) and HDL (*decreased*) in the sampled population. HDL biological activity is highly variable. It plays a crucial role in efflux of cholesterol from the peripheral cells and further reverses the cholesterol transfer from peripheral cells to the liver. HDL also neutralizes the oxidized lipids thereby acting as an antioxidant. In case of diabetes, HDL loses its antiatherogenic activity. In accordance with the previous studies,^7^, ^24^ we found that the mean HDL was reduced in diabetes population.^25^, ^26^

Group comparison based on HbA1c between healthy, prediabetes and diabetes group amongst high‐risk IDRS population shows a direct correlation of HbA1c with PPG, FBG, triglyceride, LDL and VLDL and inverse correlation with HDL. Other such studies based on Chandigarh residents suggest that the prevalence of diabetes is more in this population (13.6%) as compared to other regions of India.^27^, ^28^

We also found the mean age of prediabetes and diabetes was more than the healthy individuals amongst the high‐risk IDRS individuals. This is evident from previous studies explaining the early onset of diabetes (40‐60 years) in developing countries.^28^ Similarly, the mean weight of individuals with diabetes was higher than the healthy group of the high IDRS group. This suggests that with the increase in age and weight, people are more prone to diabetes.^28^ The higher risk of this population could be because of increasing age, obesity and family history. One of the underlying reasons could be physical inactivity (which is shown by high IDRS score), increased BMI in our studied population. Physical inactivity decreases insulin sensitivity which may cause diabetes.^28^ From the study, it is evident that sampled population analysed was overweight (BMI > 25) and aged. The selected population is at higher risk for developing diabetes, and this is reflected by two major previous studies.^27^, ^28^ Besides, this population is generally known for protective smoking lifestyle which makes the results unique to this population.^29^ Since IDRS and HbA1c both have been shown to diagnose patients with diabetes, there is need to correlate the two parameters in a larger cohort. IDRS is not used as per general guidelines even though it is simple and effective tool. It is also less expensive to detect diabetes/prediabetes in large populations. However, further confirmation is required with respect to the fasting and PPG estimation in order to detect diabetes or prediabetes as per ADA guidelines. Also, IDRS is considered as useful for prediction of risk of diabetes in real time as it does not measure the long‐term effect as in case of three month's blood glucose status assessed by HbA1c.

## 5 CONCLUSION

The study is unique for this population as it assesses the risk based on simple and cost‐effective tool, that is IDRS considering the HbA1c level. However, Chandigarh and Panchkula represent a small part of North West Indian population and hence there is a need to conduct such studies at large scale. Also, the lifestyle, per capita income and literacy rate in this region are high as compared to other regions of India, and most of the population belongs to urban locality that rarely smokes. Therefore, at this stage the transition from prediabetes to diabetes can be controlled. Such study can, therefore, act as an index of glycaemic control. Our findings can help the government to implement IDRS‐based HbA1c risk assessment, where the early control is possible thereby halting the transition of prediabetes to diabetes.

## CONFLICT OF INTEREST

Authors declare no conflict of interest.

## AUTHOR'S CONTRIBUTION

**SK** contributed to data collection, original writing and statistical analysis; **AA** contributed to concept of manuscript; **RN** contributed to proposal writing, study design, planning and monitoring; **NK** contributed to data collection and segregation of the data; **MSS** contributed to manuscript writing and statistical analysis; **VP** contributed to writing and editing; **DKP** contributed to data collection, **NM** contributed to data collection and monitoring; **AKS** contributed to planning, monitoring, data management and quality assurance; **HRN** contributed to vision, concept, proposal, planning, monitoring, advice, problem solving and editing.

## Supporting information

Table S1

## Data Availability

All the associated data is available within the article in the form of table/figure/supplementary file.
