# Supplementary material for: Prevalence of prediabetes, and diabetes in Chandigarh and Panchkula region based on glycated haemoglobin and Indian diabetes risk score
Source: Endocrinol Diabetes Metab. 2020 Nov 11;4(1):e00162. doi: 10.1002/edm2.162 (PMC7831224; doi:10.1002/edm2.162)
Supplement: Supplementary file 1 — Table S1 [file EDM2-4-e00162-s001.docx]

| **Variables** | **Sig.** | **Odds Ratio** | **95% Confidence Interval for Exp(B)** | |
| --- | --- | --- | --- | --- |
|  |  |  | **Lower Bound** | **Upper Bound** |
| Age | .002 | 1.081 | 1.048 | 1.116 |
| Gender | .000 | 2.133 | 1.306 | 3.486 |
| IDRS | .009 | 1.034 | 1.014 | 1.055 |
| BMI | .003 | 2.128 | 1.295 | 3.486 |
| Weight | .003 | 2.137 | 1.291 | 3.536 |
| Cholesterol | .002 | 2.205 | 1.344 | 3.618 |
| Triglyceride | .020 | 1.830 | 1.099 | 3.047 |
| LDL | .002 | 2.232 | 1.355 | 3.676 |
| VLDL | .019 | 1.854 | 1.108 | 3.101 |

**Supplementary Table 1:** Multinomial regression was adopted to check the association between HbA1c and age, gender, IDRS, BMI, weight, Cholesterol, Triglyceride, LDL,VLDL adjusted for gender.
